# Testosterone in Female Depression: A Meta-Analysis and Mendelian Randomization Study

**DOI:** 10.3390/biom11030409

**Published:** 2021-03-10

**Authors:** Dhruba Tara Maharjan, Ali Alamdar Shah Syed, Guan Ning Lin, Weihai Ying

**Affiliations:** 1 Med-X Research Institute and School of Biomedical Engineering, Shanghai Jiao Tong University, Shanghai 200030, China; x387735517@sjtu.edu.cn (D.T.M.); nickgnlin@sjtu.edu.cn (G.N.L.); 2 Bio-X Institutes, Key Laboratory for the Genetics of Developmental and Neuropsychiatric Disorders (Ministry of Education), Shanghai Jiao Tong University, 1954 Huashan Road, Shanghai 200030, China

**Keywords:** testosterone, depression, meta-analysis, mendelian randomization

## Abstract

Testosterone’s role in female depression is not well understood, with studies reporting conflicting results. Here, we use meta-analytical and Mendelian randomization techniques to determine whether serum testosterone levels differ between depressed and healthy women and whether such a relationship is casual. Our meta-analysis shows a significant association between absolute serum testosterone levels and female depression, which remains true for the premenopausal group while achieving borderline significance in the postmenopausal group. The results from our Mendelian randomization analysis failed to show any causal relationship between testosterone and depression. Our results show that women with depression do indeed display significantly different serum levels of testosterone. However, the directions of the effect of this relationship are conflicting and may be due to menopausal status. Since our Mendelian randomization analysis was insignificant, the difference in testosterone levels between healthy and depressed women is most likely a manifestation of the disease itself. Further studies could be carried out to leverage this newfound insight into better diagnostic capabilities culminating in early intervention in female depression.

## 1. Introduction

Depression is a major global health concern that afflicts approximately 6% of the population. Interestingly, the incidence of depression does not differ between high-income and low-income countries, which show a prevalence of 5.5% to 5.9%, respectively, indicating that depression incidence is independent of economic development and lifestyle [1]. Major depressive disorder (MDD) is a condition defined by persistent depressive symptoms, and studies have shown that almost one in five people will suffer from MDD during his or her lifetime [2]. Depression is one of the greatest contributors to years lived with disability and has resulted in a loss of individual productivity, placing an economic burden on a global scale [3,4]. Studies show that females are 1.5 to 3 times more likely to develop depression compared with men globally [5,6]. The worldwide incidence of patients diagnosed with depression is steadily increasing, which makes it one of the most challenging public health issues today, particularly for women [1,7].

Testosterone is a sex hormone that is well known for its role in reproduction. There is a significant difference in testosterone levels between the two sexes, with males displaying higher testosterone levels than females. In the male population, testosterone is mainly secreted by the Leydig cells of the testes, while in females, testosterone is produced by the ovaries and adrenal glands. However, the average serum testosterone levels are between 10- and 20-fold lower in females overall. Due to this difference in concentration between the genders, testosterone is usually recognized as a male sex hormone, but a commonly overlooked fact is that women are more sensitive to fluctuations in testosterone levels [8]. Several studies investigating the neuropsychiatric manifestations of testosterone have found that low serum testosterone levels may be involved in the development of depression [9,10,11] and influence emotional processing, memory, and perception [12]. Evidence from multiple studies indicates that testosterone has anxiolytic and antidepressant effects, with the potential to promote improved mood and mental health in both men and women [10,13,14].

Androgen receptors are expressed in various tissues throughout the human body, including the brain’s limbic systems [13,15], and studies have shown that changes in testosterone levels could influence a wide variety of health problems, such as impotence, osteoporosis, and muscle weakness, as well as factors contributing to mental health, including fatigue, sleep disturbances, irritability, and depressed mood [16,17,18]. Serum testosterone levels steadily decline as an individual ages in both men and women. However, the decline is more pronounced in males, and there is evidence suggesting that this reduction in testosterone levels may lead to depression in older men [19]. Hypogonadism is a clinical condition defined by serum testosterone concentrations of <250–300 ng/dL in men [20], and studies have shown a significant relationship between hypogonadism and depression in men [21]. Males undergoing androgen-depleting therapy for prostate cancer treatment also display a greater risk of developing MDD [22], while testosterone therapy appears to be effective in alleviating symptoms of depression in older hypogonadal men [10,23,24,25,26]. Furthermore, an increase in circulating levels of testosterone along with low levels of sex-hormone-binding globulin (*SHBG*) is associated with cardiovascular disease, insulin resistance, and visceral obesity [27], while cardiovascular morbidity and mortality may be correlated with major depression [28].

The effects of the menstrual cycle on mood have been well studied, and fluctuations in estrogen and progesterone levels are commonly known to influence behavior [29,30]. Testosterone has also been shown to have an effect on female behavior but is rarely recognized [31]. Ovariectomies and menopause can result in drastically lowered serum testosterone in women that may lead to depression [32], suggesting that decreased levels of testosterone may play a role in the onset of depression and antisocial behavior in women, and since females are more susceptible to depression and display greater symptom severity [33], it is surprising that studies so far have mostly focused on the relationship between testosterone and depressive behavior in men. To date, studies that have investigated the relationship between testosterone and depression in women report inconsistent results, with some studies suggesting that female depression patients have lower serum testosterone levels than healthy controls [32,34], while others report higher serum testosterone levels in female depression patients [35,36,37]. Interestingly, testosterone levels have been shown to differ among premenopausal and postmenopausal depressive patients [38].

Mendelian randomization (MR) is a powerful tool that can be used to make causal inferences in exposure–outcome relationships. Since genetic factors are assigned at birth and these factors have been significantly associated with exposures of interest, multiple genetic factors can be combined into a genetic instrument that can be used to determine whether the exposure can be causally associated with the outcome of interest. MR studies can therefore bypass most of the confounders, such as smoking, that may influence our observations and have recently become increasingly popular, as they are seen as an alternative to randomized control trials (RCTs) but almost infinitely cheaper to perform [39]. A few MR studies investigating the role of sex hormones in disease have successfully highlighted significant casual relationships; for example, higher testosterone increases the risks of type 2 diabetes and polycystic ovary syndrome in women [28] and may also result in depression in males [40]. However, the causal relationship between testosterone and depression in women is still unclear.

Taking together all of the above presents a mixed picture and raises the question of whether testosterone levels are indeed associated with depression in women, and if so, what is the direction of this association? In order to better understand the testosterone–depression relationship in the female population, here we attempt to collect all the published data concerning testosterone levels in cohorts of female depressed patients and conduct a meta-analysis and MR, which would allow us to answer the above questions and help us develop a greater understanding of the issue while potentially aiding in the treatment of depression in women. To the best of our knowledge, this study is the first meta-analysis to address the question of testosterone level in female depression.

## 2. Materials and Methods

### 2.1. Meta-Analysis

#### 2.1.1. Search Strategy

Google Scholar and the PubMed database were searched independently by the two authors without any language restrictions applied, from the earliest valid publication until 1 October 2019. The following keywords were used for the search: testosterone or androgen and depression or MDD and female or women. Additionally, the reference lists of the resulting articles and recent review papers were manually searched to identify additional available resources.

#### 2.1.2. Inclusion Criteria

The criteria for inclusion of studies were as follows: (a) studies comprised women, (b) studies investigated patients with depression or major depressive disorder (MDD), (c) testosterone levels were compared between depressed groups and controls, (d) the total testosterone level was tested in blood or serum, (e) controls who were matched for race and gender were used, and (f) diagnosis of depression was made by trained researchers or psychologists using either one of the Diagnostic and Statistical Manual of Mental Disorders edition III (DSM III-R) or later versions.

#### 2.1.3. Exclusion Criteria

The criteria for exclusion of studies were as follows: (a) studies that were restricted to the administration of testosterone therapy, (b) studies without proper control groups, (c) studies that failed to present depression diagnosis criteria, (d) studies that failed to disclose the method by which they quantified testosterone, (e) studies that investigated testosterone levels in tissues other than serum, and (f) review articles.

#### 2.1.4. Data Extraction

**Figure 1:**
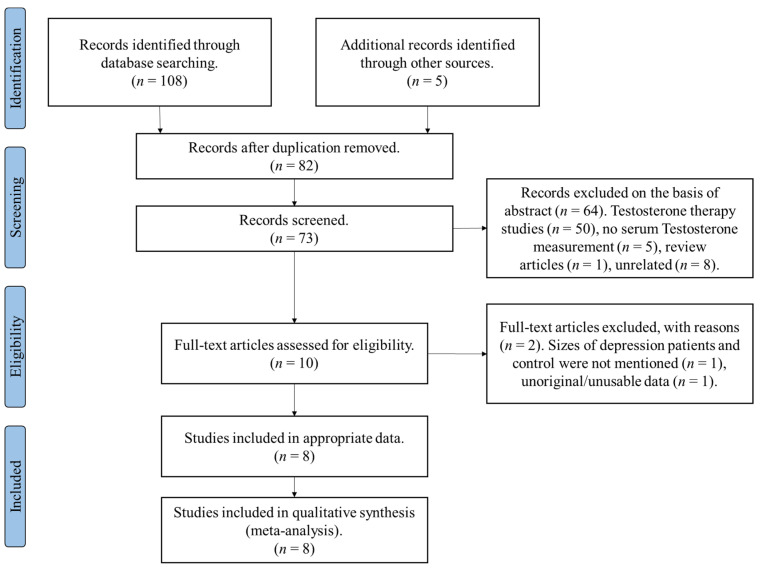
PRISMA flowchart-the study search process.

Following the completion of the literature review and screening process (Figure 1), the following variables were extracted from the selected studies: (a) author, (b) title, (c) year, (d) ethnicity, (e) MDD testosterone levels (mean, SD, sample size), (f) control testosterone levels (mean, SD, sample size), (g) depressive disorder classification, (h) testosterone quantification method, and (i) average age of patients. Data extraction was performed independently by two authors; the above data were extracted by one of the authors, and the data were verified by the other author (Table 1).

#### 2.1.5. Statistical Analysis

Meta-analyses were performed by using a random-effects model (DerSimonian–Laird method), where the standardized mean difference (SMD), also known as hedges’ adjusted *g*, was used as the effect size (ES), while studies were weighted by the continuous method. The combined effect size and heterogeneity were reported for each analysis along with their respective *p*-value. The *I*^2^ statistic was taken as a measure of heterogeneity, with *I*^2^ > 75% taken to indicate the presence of a high level and *I*^2^ < 50% taken to indicate the presence of a low level of heterogeneity. Subgroup analyses were also performed by splitting the main analyses into pre- and postmenopause subgroups. In addition to the principal analysis, further meta-analyses were performed using absolute effect size (calculated by swapping case-control data in order to flip the direction) instead of regular SMD. This analysis was predicted to be more suited for answering the question of whether testosterone levels differed significantly in depression patients independent of the direction of this effect since significant results in opposite directions would cancel out. Sensitivity analyses were performed using the leave-one-out strategy. All analyses were conducted using RevMan 5.4 (Collaboration, 2020).

### 2.2. Mendelian Randomization

In order to determine the causal effect of serum testosterone levels (exposure) on depression in women (outcome), we performed Mendelian randomization (MR) analysis using the inverse variance weighted (IVW) method for the main analysis. The genetic instrument for serum testosterone levels was created using genome wide association study (GWAS) data from the UK Biobank study [41], which comprises 425,097 individuals and is the most powerful GWAS study on testosterone to date. The resulting genetic instruments comprised variants that were significantly associated with total and bioavailable serum testosterone levels in women only. For depression (outcome), we used two different datasets: the first dataset represented a broad depression phenotype [42], and the second dataset was a meta-analysis of 807,553 MDD patients [43]. Additionally, we performed a sensitivity analysis using the weighted median, and the presence of horizontal pleiotropy was determined using MR-Egger intercept. An additional MR analysis was performed using *SHBG* as the exposure to determine whether the depressive effect was due to *SHBG*, not testosterone, since *SHBG* plays a role in the amount of bioavailable testosterone. To ensure that the variants included in the genetic instruments had no pairwise correlation with each other, significant single nucleotide polymorphisms (SNPs) were filtered using a stringent linkage disequilibrium (LD) cutoff of 0.01. Filtering, harmonization, and MR analysis were performed using the TwoSampleMR package in R.

## 3. Results

### 3.1. Meta-Analysis

#### 3.1.1. Included Studies and Participant Details

A total number of eight studies qualified for inclusion in this meta-analysis (Figure 1) (Table 1). All of the included studies studied relative serum testosterone levels between depressed female patients and healthy control groups. The meta-analyses comprised studies originating from the following populations: European (three studies), Turkish (two studies), Japanese (one study), and Indian (one study). The studies’ cohorts were either postmenopausal female depression patients or premenopausal female depression patients or a mixture of elder and premenopausal female depression patients. The studies comprised a total number of 889 participants (521 depression cases and 368 control).

**Table 1:** Characteristics of the involved studies.

Study	Year	Ethnicity	Menopause Status	Case/Con	Age (Se)
Baischer [36]	1994	European	Premenopausal	20/10	32.5 (11)
Weber [37]	2000	European	${\mathrm{Mixed}\;}^{*}$	11/11	48.1 (18)
Erdinçler [44]	2004	Turkish	Postmenopausal	34/53	70 (7.7)
Matsuzaka [45]	2004	Japanese	${\mathrm{Mixed}\;}^{*}$	44/78	53.1 (23)
Kumsar [32]	2013	Turkish	Premenopausal	52/30	31.9 (7.3)
Aswathi [35]	2015	Indian	Premenopausal	81/41	23.2 (2.7)
Giltay [46]	2017	European	Postmenopausal	230/73	70.9 (7.3)
Oulis [47]	2014	European	${\mathrm{Mixed}\;}^{*}$	38/65	54.7 (13.4)

* Mixed is defined as a study comprising women of both premenopausal and postmenopausal types.

#### 3.1.2. A Meta-Analysis of Overall Testosterone Levels

**Figure 2:**
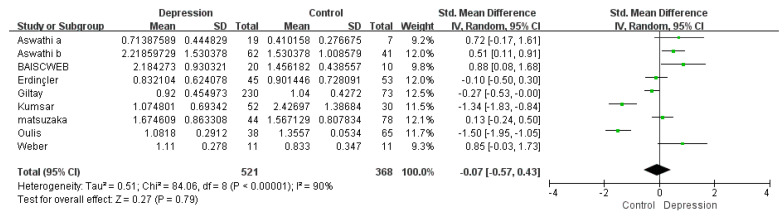
Forest plot: overall meta-analysis result-comparison of serum testosterone in depressed women vs. controls.

The overall analysis consisted of the eight studies included in the meta-analysis (*n* = 889, depression = 521, control = 368). Four out of these nine studies reported a decrease, while five reported an increase in testosterone levels in depressed women compared with controls. The result failed to show a significant difference in serum testosterone levels of depression patients vs. controls (Z = 0.27 and *p* = 0.79), while high amounts of between-study heterogeneity was observed (*I*^2^~90%, *p* < 0.00001) (Figure 2). The ES remained insignificant, and the heterogeneity remained significant throughout the sensitivity analysis.

#### 3.1.3. Subgroup Meta-Analysis

**Figure 3:**
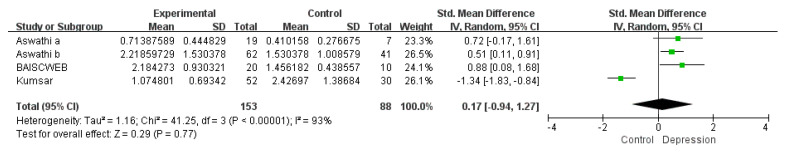
Forest plot: premenopausal subgroup meta-analysis result-comparison of serum testosterone in premenopausal depressed women vs. controls.

The premenopausal depression subgroup meta-analysis comprised three studies with a total of 241 subjects. The results failed to indicate the presence of a significant difference in testosterone levels in premenopausal depression patients compared with healthy controls (Z = 0.29, *p* = 0.77), which is consistent with the overall meta-analysis result, while significant between-study heterogeneity was also observed ($I^{2}$ = 93%, *p* < 0.00001) (Figure 3). The study by Kumsar et al. had a disproportional effect on the outcome of this analysis. When Kumsar’s study was removed from our sensitivity analysis, the heterogeneity disappeared ($I^{2}$ = 0%, *p* = 0.69), and the effect size became significant (Z = 3.55, *p* = 0.0004).

**Figure 4:**
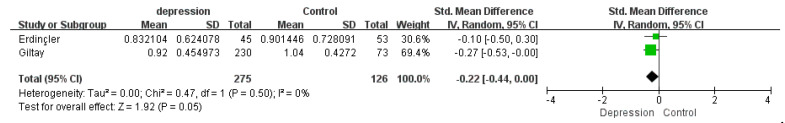
Forest plot: postmenopausal subgroup meta-analysis result-comparison of serum testosterone in postmenopausal depressed women vs. controls.

The postmenopausal depression subgroup meta-analysis comprised two studies consisting of 401 subjects. The results showed that testosterone levels were decreased (borderline significant) in postmenopausal depression patients compared with healthy controls (Z = 1.92, *p* = 0.5), while between-study heterogeneity was totally absent ($I^{2}$ = 0%, and *p* = 0.05) (Figure 4). Sensitivity analysis could not be performed because this group only comprised two studies, which were insufficient for leave-one-out analysis.

Three studies could not be included in either subgroup since they contained patients from both pre- and postmenopausal women.

#### 3.1.4. Absolute SMD Meta-Analysis

**Figure 5:**
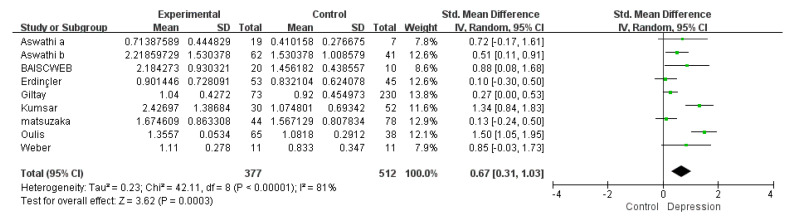
Forest plot: absolute difference overall meta-analysis result-comparing serum testosterone in depressed women vs. controls.

The overall meta-analysis was repeated using absolute SMD instead of simple SMD as the effect size. The results of this meta-analysis showed a difference in serum testosterone levels in depressed women compared with controls (Z = 3.62 and *p* = 0.0003) (Figure 5). Between studies, heterogeneity was also reduced from 90% to 81%. Both between-study heterogeneity and effect size remained significant throughout the sensitivity analysis.

**Figure 6:**
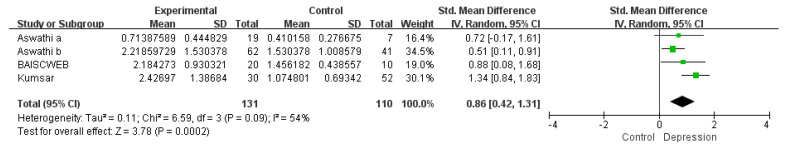
Forest plot: premenopausal subgroup absolute difference meta-analysis result-comparison of serum testosterone in premenopausal depressed women vs. controls.

Only premenopausal depression subgroup meta-analysis was performed since the postmenopausal subgroup comprised studies that already had the same direction of effect. Results from the meta-analysis also showed a significant difference in testosterone levels of depressed premenopausal women compared with controls (Z = 3.78, *p* = 0.0002) (Figure 6), while also reporting a reduction in between-study heterogeneity from 93% to 54% compared with the regular meta-analysis. Removal of Kumar et al. or Aswathi et al. during the sensitivity analysis resulted in eliminating the between-study heterogeneity without changing the significance of the effect size.

### 3.2. Mendelian Randomization

Mendelian randomization of genetically predicted testosterone, both total and bioavailable, with depression datasets failed to show any significant association between testosterone in women and depression-related traits. The result remained true for the relationship between *SHBG* and depression and all weight median MR analyses. The result indicates that increased or decreased testosterone does not cause depression (Table 2, Table 3 and Table 4).

## 4. Discussion

Currently, the clinical diagnosis of depression is made through the DSM-V or ICD10 manuals standards, and depression remains severely underdiagnosed [48,49]. Only those who manifest severe symptoms are likely to seek aid. To achieve early diagnosis and intervention of depression, potential biomarkers of the disease need to be identified. Our study investigated testosterone’s role and attempted to determine whether depressed women exhibit a difference in serum testosterone levels by conducting a meta-analysis. A total of eight studies were deemed eligible for inclusion in our meta-analysis. However, these studies differed from each other quite significantly in terms of average age, ethnicity, and other factors. In order to circumvent these confounding factors, we supplemented our meta-analysis with a Mendelian randomization study. To the best of our knowledge, this study is the first to perform such a meta-analysis.

Our meta-analysis suggests that there is a significant deviation in testosterone levels in depressed women compared with nondepressed women. However, the direction of these differences can often differ. A meta-analysis of the premenopausal subgroup showed an overall increase in testosterone levels, while the postmenopausal subgroup exhibited lower testosterone levels than nondepressed women of similar age. However, we do believe that the effects are opposite based on whether a woman has experienced menopause or because testosterone itself may be a symptom of depression and does not cause depression by itself.

In order to determine whether the fluctuations in testosterone levels could be the cause of depression in women, we performed Mendelian randomization analyses using multiple, most recent datasets. However, all of our MR analyses, which utilized genetic instruments for total and free testosterone and *SHBG* in two independent datasets of patients suffering from MDD and broad depression, failed to show any significant causal relationship between genetically predicted serum testosterone and depression. As a result of both our Mendelian randomization and meta-analyses, we can state that testosterone does not play a causal role in female depression. It is most likely that the discrepancies observed in testosterone levels are a result of the disease itself. More research may be required to uncover the underlying mechanism of how depression could lead to an increase or decrease in testosterone levels. It is also possible that the relationship between testosterone levels and depression in women may be parabolic. For example, the deviation of testosterone levels from an optimum range would result in adverse effects, and there are even some studies that support this theory [50,51].

Recent studies have proved that testosterone replacement therapy does indeed have antidepressive effects in men [14], and our results highlight the fact that a similar disruption of testosterone levels exists in females. Testosterone replacement therapy could potentially aid in the treatment of depressed women, but larger observational studies of the testosterone levels in female depressed patients, followed by testosterone therapy trials, would need to be performed in the future before testosterone could be used to diagnose and treat depression in women.

The major limitation of our study was the number of studies that focused on depression in postmenopausal women, as our meta-analysis of this subgroup comprised only two studies. Another limitation of our investigation was that the MR analyses were performed using genetic instruments comprising SNPs significantly associated with testosterone levels in women. However, the outcome GWAS of depression was made up of both genders. The MR analyses could have been improved if the outcome GWAS was also female only. Additionally, there was a high amount of heterogeneity observed in our overall meta-analysis. As expected, the use of absolute SMD resulted in a reduction of between-study heterogeneity, which we believe justifies using this methodology. Five of the eight studies included in our meta-analysis reported significant results. Combining them using simple SMD may have been inappropriate since two studies reporting significant results, albeit with opposing directions of effect, would yield a null result. That being said, our results calculated by using absolute SMD in meta-analysis showed that there is a significant difference in testosterone levels in depressed women without shedding knowledge of the direction of effect. The use of absolute SMD resolved some but not all of the between-study heterogeneity observed in our analyses. The presence of heterogeneity could be because the meta-analysis consisted of patients from four different ethnicities while comprising only eight studies. Another probable cause of the heterogeneity observed throughout our analysis were the different methods employed to quantify testosterone in serum.

## 5. Conclusions

In conclusion, our results show that testosterone levels are significantly altered in female depression and suggest that testosterone may have utility as a biomarker for depression, particularly in postmenopausal depression. Our study sheds light on testosterone’s role in female depression and provides direction for future research in the area. Further research into testosterone’s role in depression may lead to earlier diagnosis and intervention of the disorder, undoubtedly leading to better clinical outcomes.

## Figures and Tables

**Table 2:** The effect of genetically predicted total testosterone on depression in women.

Outcome	SNPs	IVW (*p*-Val.)	WM (*p*-Val.)	MR-Egger Intercept (*p*-Val.)
Broad depression	125	0.005 (0.20)	0.011 (0.08)	0.43
MDD	121	0.0216 (0.22)	0.013	0.70

Inverse variance weighted (IVW) and weighted median (WM) report beta coefficients and *p*-values from Mendelian randomization analysis of total serum testosterone and depression using IVW and WM methods. The IVW method is used in the main analysis, while WM and MR-Egger intercept are used in the sensitivity analysis.

**Table 3:** The effect of genetically predicted bioavailable testosterone on depression in women.

Outcome	SNPs	IVW (*p*-Val.)	WM (*p*-Val.)	MR-Egger Intercept (*p*-Val.)
Broad depression	92	0.0005 (0.94)	0.0005 (0.95)	0.80
MDD	91	0.0028 (0.92)	0.006 (0.86)	0.89

Inverse variance weighted (IVW) and weighted median (WM) report beta coefficients and *p*-values from Mendelian randomization analysis of bioavailable serum testosterone and depression using IVW and WM methods. The IVW method is used in the main analysis, while WM and MR-Egger intercept are used in the sensitivity analysis.

**Table 4:** The effect of genetically predicted serum *SHBG* on depression in women.

Outcome	SNPs	IVW (*p*-Val.)	WM (*p*-Val.)	MR-Egger Intercept (*p*-Val.)
Broad depression	170	0.009 (0.35)	0.001 (0.93)	0.18
MDD	173	0.043 (0.249)	0.0003 (0.99)	0.67

Inverse variance weighted (IVW) and weighted median (WM) beta coefficients and *p*-values from Mendelian randomization analysis of serum *SHBG* protein and depression using IVW and WM methods. The IVW method is used in the main analysis, while WM and MR-Egger intercept are used in the sensitivity analysis.

## Data Availability

Not applicable.
